# Cervical squamous cell carcinoma-secreted exosomal miR-221-3p promotes lymphangiogenesis and lymphatic metastasis by targeting VASH1

**DOI:** 10.1038/s41388-018-0511-x

**Published:** 2018-09-25

**Authors:** Chen-Fei Zhou, Jing Ma, Lei Huang, Hong-Yan Yi, Yan-Mei Zhang, Xiang-Guang Wu, Rui-Ming Yan, Li Liang, Mei Zhong, Yan-Hong Yu, Sha Wu, Wei Wang

**Affiliations:** 1 Department of Obstetrics and Gynecology, The First Affiliated Hospital of Guangzhou Medical University, Guangzhou, 510120 China; 2 Department of Obstetrics and Gynecology, Nanfang Hospital/The First School of Clinical Medicine, Southern Medical University, Guangzhou, 510515 China; 3 Institute of Cellular Medicine, Faculty of Medical Sciences, Framlington Place, Newcastle University, Newcastle-Upon-Tyne, NE2 4HH UK; 4 Department of Immunology, School of Basic Medical Sciences, Southern Medical University, Guangdong Provincial Key Laboratory of Proteomic, Guangzhou, 510515 China; 5 Department of Pathology, School of Basic Medical Sciences, Southern Medical University, Guangzhou, 510515 China

**Keywords:** Cervical cancer, Metastasis

## Abstract

Cancer-secreted exosomal miRNAs are emerging mediators of cancer-stromal cross-talk in the tumor environment. Our previous miRNAs array of cervical squamous cell carcinoma (CSCC) clinical specimens identified upregulation of miR-221-3p. Here, we show that miR-221-3p is closely correlated with peritumoral lymphangiogenesis and lymph node (LN) metastasis in CSCC. More importantly, miR-221-3p is characteristically enriched in and transferred by CSCC-secreted exosomes into human lymphatic endothelial cells (HLECs) to promote HLECs migration and tube formation in vitro, and facilitate lymphangiogenesis and LN metastasis in vivo according to both gain-of-function and loss-of-function experiments. Furthermore, we identify vasohibin-1 (VASH1) as a novel direct target of miR-221-3p through bioinformatic target prediction and luciferase reporter assay. Re-expression and knockdown of VASH1 could respectively rescue and simulate the effects induced by exosomal miR-221-3p. Importantly, the miR-221-3p-VASH1 axis activates the ERK/AKT pathway in HLECs independent of VEGF-C. Finally, circulating exosomal miR-221-3p levels also have biological function in promoting HLECs sprouting in vitro and are closely associated with tumor miR-221-3p expression, lymphatic VASH1 expression, lymphangiogenesis, and LN metastasis in CSCC patients. In conclusion, CSCC-secreted exosomal miR-221-3p transfers into HLECs to promote lymphangiogenesis and lymphatic metastasis via downregulation of VASH1 and may represent a novel diagnostic biomarker and therapeutic target for metastatic CSCC patients in early stages.

## Introduction

Cervical squamous cell carcinoma (CSCC) is one of the most prevalent malignancies, and its incidence in female malignancies worldwide is ~ 15% [1]. Although the combination of screening and surgery has effectively improved the prognosis of early-stage CSCC, it is difficult to completely prevent metastasis and recurrence of CSCC, which is the leading cause of women’s death from this disease [2]. As the major spreading route, lymphatic metastasis is an independent risk factor for clinical outcomes of early-stage CSCC [3]. More than 20% of patients with early-stage CSCC suffer from postoperative recurrence, largely owing to the occurrence of lymphatic metastasis prior to surgery [4, 5].

Among multiple factors underlying lymphatic metastasis, the adaptation of the primary tumor microenvironment by cancer to facilitate tumor cell dissemination plays an important prometastatic role [6]. Lymphangiogenesis is the process of growing new lymphatic vessels and correlates with the incidence of lymphatic metastasis and poor prognosis in multiple cancers [7–9]. Growing evidence revealed that lymphatic vessels in the tumor periphery served as a highway for tumor cells to disseminate from their primary site to regional lymph nodes (LNs) [10, 11]. However, the molecular mechanism of tumor-driven peritumoral lymphangiogenesis is not well defined.

miRNAs are small non-coding RNAs that pair to 3′-untranslated regions (UTRs) of target mRNA, resulting in mRNA destabilization and/or posttranscriptional suppression [12]. The biosynthesis and dysregulation of various miRNAs is closely associated with cancer progression [13]. We have recently performed miRNA array in paired CSCC tissues and identified upregulation of miR-221-3p [14]. Although previously considered to be only in cells, miRNAs have also been reported to be present extracellularly as a major RNA component of exosomes [15]. Exosomes are small, 30–100 nm membrane vesicles that are secreted into the extracellular environment by multiple cell types, including cancer cells [16]. Cancer-secreted exosomal miRNAs can be transferred into recipient normal host cells, regulating target genes, and thus regulate biological processes in localized tumors as well as distal tissues [17, 18]. Exosomal miRNAs reflect the expression patterns of dysregulated miRNAs in cancer cells to a certain extent [19]. Therefore, cancer-secreted exosomal miRNAs are important regulatory molecules in mediating cancer–host cross-talk.

In this study, miR-221-3p was closely correlated with peritumoral lymphangiogenesis and LN metastasis. Moreover, it was also highly enriched in exosomes secreted from CSCC cell lines compared with non-carcinoma epithelial cell line. Although our previous study has reported that miR-221-3p enhanced the malignancy of CSCC cells [14], its roles in lymphangiogenesis and lymphatic metastasis of CSCC need to be further investigated.

To address this problem, we performed the current study to investigate the underlying molecular mechanisms for cancer-secreted exosomal miR-221-3p in regulating lymphangiogenesis and lymphatic metastasis in CSCC, as well as its clinical relevance, to explore the potential clinical applications in diagnosis and therapy.

## Results

### Upregulation of miR-221-3p positively correlates with LN metastasis of CSCC

**Fig. 1:**
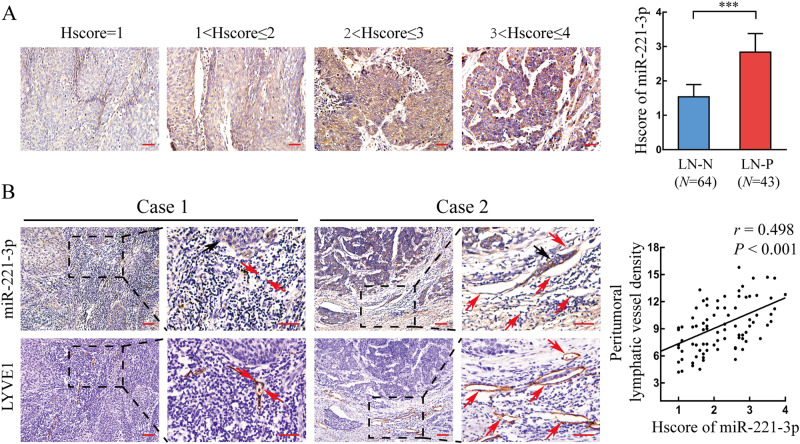
Upregulation of miR-221-3p in the primary tumor positively correlates with lymph node metastasis of CSCC. **a** ISH scores (HSCORE) of miR-221-3p were analyzed between the LN-N (LN negative; *n* = 64) and LN-P (LN positive; *n* = 43) groups in CSCC specimens. **b** Staining of miR-221-3p and LYVE1 (lymphatic marker) in serial sections of CSCC specimens. Representative micrographs are shown (left). The tumor cells are indicated by black arrows. The lymphatic vessels are indicated by red arrows. Correlations between miR-221-3p staining and peritumoral lymphatic vessel density (PLVD) were analyzed (right). Scale bar, 50 µm. Error bars represent the mean ± SD of three independent experiments. ***, *P* < 0.001

To identify the correlation between miR-221-3p levels and LN metastasis of CSCC, miR-221-3p levels were examined in 107 paraffin-embedded human CSCC serial sections using in situ hybridization (ISH). Compared with the LN-negative group, a significantly high level of miR-221-3p was detected at the primary tumor site in the LN-positive group (Fig. 1a). Correspondingly, higher miR-221-3p levels strongly correlated with the increment of peritumoral lymphatic vessel density (PLVD) in serial sections of CSCC specimens, as indicated by LYVE1-positive vessels using immunohistochemistry (IHC) (*r* = 0.498, *P* < 0.001) (Fig. 1b). More importantly, as shown in Fig. 1b, in addition to tumor cells, high miR-221-3p levels were also present in some peritumoral lymphatic vessels. Taken together, these results suggested that high levels of miR-221-3p expression may promote lymphangiogenesis and facilitate lymphatic metastasis in CSCC.

### miR-221-3p can be enriched in CSCC-secreted exosomes and transferred to LECs

**Fig. 2:**
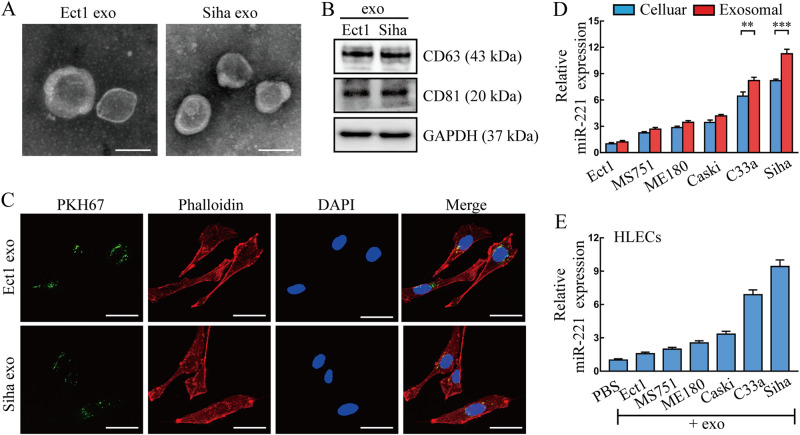
miR-221-3p can be enriched in CSCC-secreted exosomes and transferred to HLECs. **a** Morphology of Ect1 and Siha-secreted exosomes was confirmed by transmission electron microscopy. Scale bar, 50 nm. **b** Positive markers (CD63 and CD81) of Ect1 and Siha-secreted exosomes were detected by western blot. **c** Human lymphatic endothelial cells (HLECs) pre-treated with PKH67-labeled exosomes secreted by Ect1 and Siha for 48 h were stained by phalloidin (red) and DAPI (blue) for confocal microscopy analysis. Scale bar, 20 µm. **d** Basic miR-221-3p levels in indicated cells and paired exosomes were detected by qRT-PCR. **e** miR-221-3p levels in HLECs pre-treated with PBS or indicated exosomes for 24 h were detected by qRT-PCR. Exo, exosomes. miR-221, miR-221-3p. Error bars represent the mean ± SD of three independent experiments

Previous studies have suggested that miRNAs could be transferred intercellularly by exosomes [20]. Therefore, we investigated whether exosomes were also involved in miR-221-3p delivery from CSCC cells to human lymphatic endothelial cells (HLECs). Exosomes were initially purified from the supernatant of Siha and Ect1 cells. Typical cup-shaped morphology and a size range of 30–100 nm was confirmed by transmission electron microscopy (Fig. 2a). The exosomal positive markers including CD63 and CD81 were detected by western blot (Fig. 2b). Purified exosomes were then labeled with a fluorescent membrane tracer PKH67 (green) and incubated with human lymphatic endothelial cells (HLECs) and mouse lymphatic endothelial cells (MLECs). After 48 h incubation, exosome fused cells were stained by phalloidin (red) and 4′,6-diamidino-2-phenylindole (blue) for confocal microscopy evaluation. A green fluorescent punctuating signal inside the cytoplasm of recipient HLECs indicated the internalization of exosomes (Fig. 2c). The same phenomenon was also observed in MLECs incubated with PKH67-labeled exosomes (Fig. S1A).

Furthermore, we found that miR-221-3p was enriched in CSCC-secreted exosomes, especially from C33a and Siha (Fig. 2d), relative to their cellular content and compared with exosomes secreted by non-carcinoma epithelial (Ect1). To confirm that CSCC-secreted miR-221-3p can be transferred to HLECs and MLECs via exosomes, we measured the miR-221-3p levels in HLECs and MLECs pre-treated with CSCC-secreted exosomes. An increase of the cellular levels of miR-221-3p was observed in recipient HLECs and MLECs following treatment with CSCC-secreted exosomes (Fig. 2e and Fig. S1B). Taken together, these results suggest that horizontal transfer of miRNA-221-3p from CSCC cells to LECs can be performed cross-species via exosomes.

### CSCC-secreted exosomal miR-221-3p promotes lymphangiogenesis in vitro

**Fig. 3:**
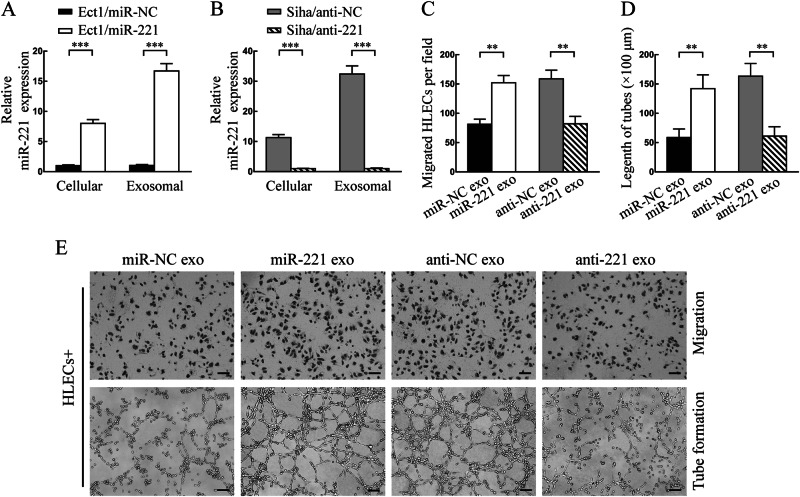
CSCC-secreted exosomal miR-221-3p promotes lymphangiogenesis in vitro. **a** Cellular and exosomal miR-221-3p levels in Ect1 stably transfected with miR-221-3p overexpression (miR-221) or negative control (miR-NC) lentivectors were detected by qRT-PCR. **b** Cellular and exosomal miR-221-3p levels in Siha stably transfected with miR-221-3p knockdown (anti-221) or negative control (anti-NC) lentivectors were detected by qRT-PCR. **c** Transwell migration assay in HLECs pre-treated with indicated exosomes. Average migrated cells per field were calculated. **d** Tube formation assay in HLECs pre-treated with indicated exosomes. Average length of tubes per field were calculated. **e** Representative micrographs of migration (upper panel) and tube formation assay (lower panel) in HLECs pre-treated with indicated exosomes are shown. Scale bar, upper panel, 50 µm; lower panel, 100 µm. miR-NC exo, Ect1/miR-NC secreted exosomes. miR-221 exo, Ect1/miR-221-3p secreted exosomes. anti-NC exo, Siha/anti-NC secreted exosomes. anti-221 exo, Siha/anti-221-3p secreted exosomes. Error bars represent the mean ± SD of three independent experiments. **, *P* < 0.01; ***, *P* < 0.001

To investigate the role of exosomal miR-221-3p in lymphangiogenesis, lentiviral vector overexpressing miR-221-3p or negative control (NC) was transfected into Ect1 (low miR-221-3p), and the lentiviral vector silencing miR-221-3p or NC was applied to Siha (high miR-221-3p). Quantitative reverse transcriptase-polymerase chain reaction (qRT-PCR) analysis showed that cellular and exosomal miR-221-3p levels were significantly higher in of Ect1/miR-221-3p and Siha/anti-NC compared with Ect1/miR-NC and Siha/anti-221-3p (Fig. 3a, b). Then, HLECs and MLECs incubated with exosomes secreted by Ect1/miR-NC (low miR-221-3p), Ect1/miR-221-3p (high miR-221-3p), Siha/anti-NC (high miR-221-3p), and Siha/anti-221-3p (low miR-221-3p) for 48 h were analyzed for proliferation, migration, and tube formation assays. The results showed that exosomes with high miR-221-3p dramatically promoted HLECs and MLECs migration and tube formation compared with those with low miR-221-3p (Fig. 3c–e and Fig. S2C–E). Interestingly, proliferation of HLECs and MLECs seemed to not be affected by exosomes with high or low miR-221-3p (Fig. S2A–B). Collectively, these results suggest that exosomal miR-221-3p promotes lymphangiogenesis in vitro.

### CSCC-secreted exosomal miR-221-3p promotes lymphangiogenesis and lymphatic metastasis in vivo

The effect of exosomal miR-221-3p on CSCC lymphangiogenesis and lymphatic metastasis was assessed in vivo using a popliteal LN metastasis model. We first randomly established footpad xenografts using mCherry-labeled Siha/anti-221-3p or Siha/anti-NC cells (*n* = 3/group, repeated twice). Tumor sizes in Siha/anti-221-3p group were much smaller and expressed lower levels of miR-221-3p comparing with the Siha/anti-NC group (Fig. S3A–B). To exclude the effect of different tumor size on LN metastasis [21], popliteal LNs were harvested and analyzed IHC for mCherry expression when the primary tumors reached ~ 150 mm^3^. We found that Siha/anti-221-3p group had non-metastatic popliteal LNs compared with Siha/anti-NC group (Fig. S3C).

**Fig. 4:**
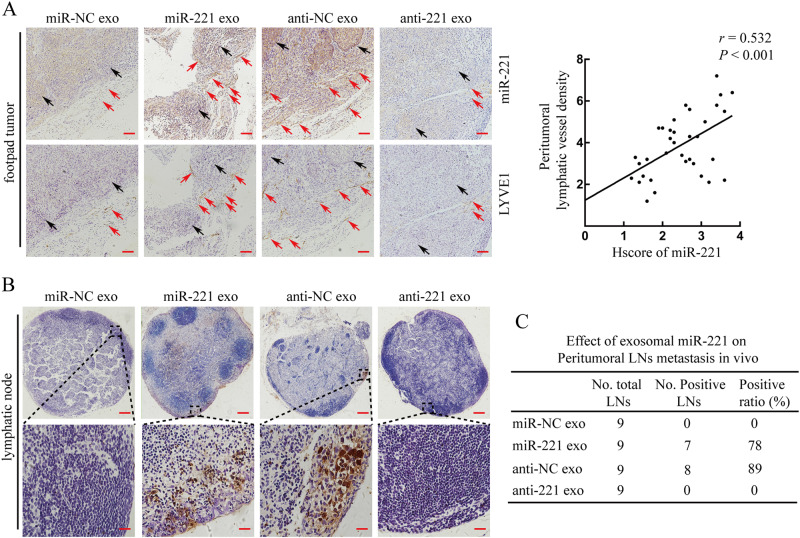
CSCC-secreted exosomal miR-221-3p promotes lymphangiogenesis and lymphatic metastasis in vivo. **a** popliteal lymph node metastasis model was established in nude mice by inoculating the footpad with Siha/anti-221-3p (5 × 10^6^) stably expressing mCherry. When footpad tumor size reached 50 mm^3^, exosomes (10 µg) secreted by Ect1/miR-NC, Ect1/miR-221-3p, Siha/anti-NC, or Siha/anti-221-3p were then injected into the center of the tumors (*n* = 3/group, repeated twice) twice a week. After five injections, primary tumors reached a comparable size of ~ 150 mm^3^, and then footpad tumors and popliteal LNs were collected for study. **a** Staining of miR-221-3p and LYVE1 in serial sections of mice footpad tumors. Representative micrographs of positive staining are shown (left). The tumor cells are indicated by black arrows. The lymphatic vessels are indicated by red arrows. The correlation between miR-221-3p levels and PLVD was statistically analyzed (right). Scale bar, 20 µm. **b** Staining of mCherry in popliteal LNs from mice treated with the indicated exosomes. Representative micrographs are shown. Metastasis-positive LNs were identified by staining for cancer cell-expressed mCherry. Scale bar, upper panel, 200 µm; lower panel, 20 µm. **c** The ratio of metastasis-positive to total dissected popliteal LNs from mice treated with the indicated exosomes. ***, *P* < 0.001

According to the above data, we further inoculated mCherry-labeled Siha/anti-221-3p cells into the footpads of nude mice. At the time point when the tumor size reached 50 mm^3^, exosomes secreted by Ect1/miR-NC, Ect1/miR-221-3p, Siha/anti-NC, or Siha/anti-221-3p were then randomly injected into the center of the xenograft tumors (*n* = 3/group, repeated twice) twice a week. When the primary tumors grew to ~ 150 mm^3^ after five injections, we killed the mice and harvested the tumors and the associated popliteal LNs for ISH and IHC analysis. A significantly higher level of miR-221-3p was both present in some peritumoral lymphatic vessels and tumor cells treated exosomes with high miR-221-3p secreted from Ect1/miR-221-3p and Siha/anti-NC compared with those with low miR-221-3p secreted from Ect1/miR-NC and Siha/anti-221-3p (Fig. 4a). Meanwhile, we performed qRT-PCR analysis for miR-221-3p expression and conformed ISH results (Fig. S4A–B). Furthermore, there was a strong positive correlation between miR-221-3p expression and PLVD (Fig. 4a). In addition, a higher ratio of metastasis-positive popliteal LNs was found in groups with high exosomal miR-221-3p compared with those with low exosomal miR-221-3p (Fig. 4b, c). Interestingly, although tumor size was larger in exosomes with high miR-221-3p groups (Fig. S5A), there were no significant differences in micro-vessel density for the comparable tumor sizes after treatment with exosomes with high or low miR-221-3p groups (Fig. S5B). Taken together, these results indicate that CSCC-secreted exosomal miR-221-3p could promote lymphangiogenesis and LN metastasis in vivo.

### CSCC-secreted exosomal miR-221-3p targets lymphatic VASH1 to induce lymphangiogenesis

**Fig. 5:**
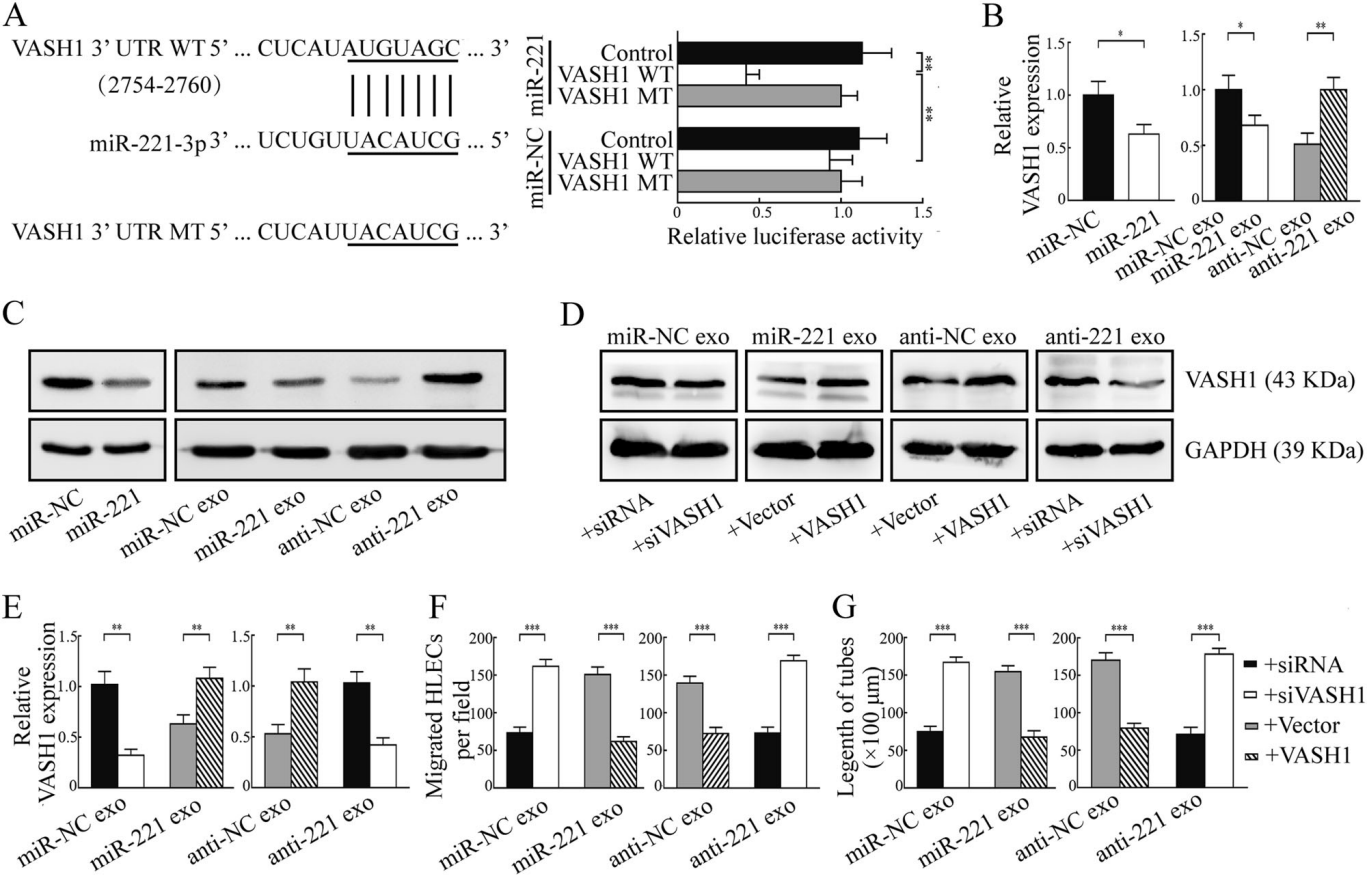
CSCC-secreted exosomal miR-221-3p targets lymphatic VASH1 to induce lymphangiogenesis in HLECs. **a** RNA sequence alignment between miR-221-3p and the 3′-UTR of VASH1 (left), and the effect of miR-NC and miR-221-3p on the activity of the luciferase reporter containing either wild type (WT) or mutant type (MT) were tested by dual-luciferase reporter assay (right). **b**–**c** RNA and protein levels of VASH1 were, respectively, detected by qRT-PCR and western blot in HLECs transfected with miR-221-3p mimic or negative control (NC) compared with those treated with the indicated exosomes. **d**–**e** RNA and protein levels of VASH1 were, respectively, detected by qRT-PCR and western blot in HLECs treated with indicated exosomes in the presence of VASH1 overexpression plasmid (VASH1) or vector control and VASH1 silence fragment (siVASH1) or siRNA control. **f**–**g** Overexpression of VASH1 rescued the biologic effects associated with exosomal miR-221-3p, whereas knockdown of VASH1 simulated the biologic effects associated with exosomal miR-221-3p through cell migration and tube formation assays. Error bars represent the mean ± SD of three independent experiments. *, *P* < 0.05; **, *P* < 0.01; ***, *P* < 0.001

Multiple algorithms (miRWalk, PicTar, and TargetScan) were used to identify the candidate targets of miR-221-3p. As a result, 135 target genes were predicted to be regulated by miR-221-3p (Table S4). Among these candidates, vasohibin-1 (VASH1) functioned as a negative regulator of lymphangiogenesis confirmed by Gene Ontology Consortium (Table S5) and was chosen for further study [22]. To examine miR-221-3p regulation of the putative target VASH1, the predicted miR-221-3p-binding site in the 3′-UTR of VASH1 (wild type) or the mutated sequence (mutant type) were cloned into luciferase reporter plasmids and assessed for their response to miR-221-3p in HLECs. The results showed that the expression of the reporter gene followed by a wild type 3′-UTR of VASH1 was significantly reduced by the co-transfected miR-221-3p mimic, whereas reporter gene expression had no change if followed by the 3′-UTR of the VASH1 gene with a mutated putative target site of miR-221-3p (Fig. 5a). Thus, we concluded that VASH1 is a direct target of miR-221-3p.

Consistent with the results from the luciferase reporter assay, treatment with miR-221-3p mimic or exosomes derived from Ect1/miR-221-3p and Siha/anti-NC but not the Ect1/miR-NC and Siha/anti-221-3p resulted in a significant decrease of VASH1 expression in HLECs for both RNA and protein levels (Fig. 5b, c). Re-expression VASH1 rescued, whereas silencing VASH1-simulated downregulation of VASH1 mediated by exosomes with high levels of miR-221-3p (Fig. 5d, e).

Further in vitro studies confirmed that re-expression of VASH1 could abrogate Ect1/miR-221-3p and Siha/anti-NC exosomes-mediated promotion of migration and tube formation of HLECs, whereas silencing VASH1 could provoke Ect1/miR-NC and Siha/anti-221-3p exosomes' abilities to induce migration and tube formation in HLECs (Fig. 5f, g and Fig. S6A–B). Collectively, these results indicated that exosomal miR-221-3p induced lymphangiogenesis via suppressing VASH1 expression in HLECs.

Growing evidence has suggested that the ERK and AKT pathways are involved in lymphangiogenesis and lymphatic metastasis [23], which prompted us to determine whether the ERK and AKT pathways could be activated by exosomal miR-221-3p. We found that phosphorylation of ERK1/2 and AKT was significantly increased in exosomes with high miR-221-3p groups rather than those with low miR-221-3p groups (Fig. S7A). In addition, as a classic lymphangiogenic growth factor, tumor-derived VEGF-C could also induce lymphangiogenesis via activation of the ERK and AKT signaling pathways in HLECs [24], and the relationship between miR-221-3p and VEGF-C expression was further examined in CSCC. Interestingly, qRT-PCR and ELISA showed that miR-221-3p overexpression in Ect1 or miR-221-3p knockdown in Siha did not induce VEGF-C mRNA or protein changes (Fig. S7B). Therefore, we concluded that the miR-221-3p-VASH1 axis activated AKT and ERK signaling pathways might work in a VEGF-C independent manner in HLECs.

### CSCC-secreted exosomal miR-221-3p is associated with VASH1 expression and lymphatic metastasis

**Fig. 6:**
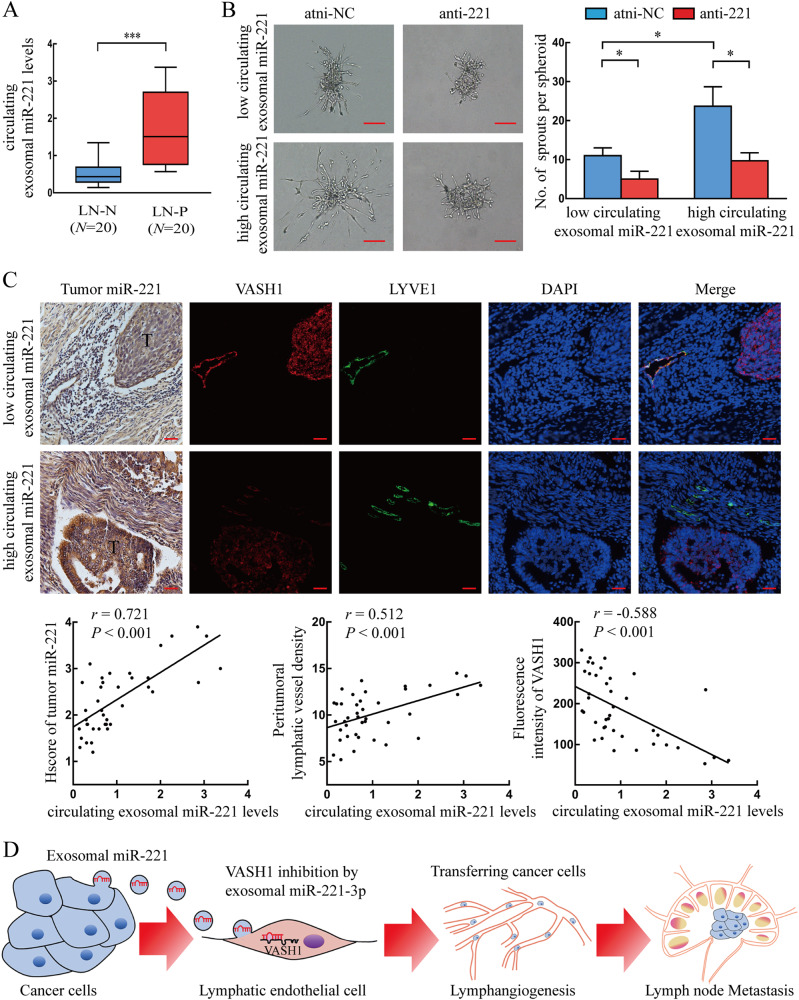
CSCC-secreted exosomal miR-221-3p is associated with VASH1 expression and lymphatic metastasis. **a** miR-221-3p levels in circulating exosomes of CSCC patients with LN-N (LNs negative; *n* = 20) and LN-P (LNs positive; *n* = 20) were detected by qRT-PCR. **b** Sprout assay in HLECs pre-treated with circulating exosomes from LN-N patients with low miR-221-3p levels or LN-P patients with high miR-221-3p levels in the presence of miR-221-3p inhibitor (anti-221) or negative control (NC). Representative micrographs are shown (left). Average sprouts per spheroid were calculated (right). Scale bar, 50 µm. **c** Correlation analyses of exosomal miR-221-3p, tumor miR-221-3p, peritumoral lymphatic vessel density and VASH1 levels in the above CSCC patients. CSCC specimens subjected to ISH for tumor miR-221-3p and double-label IF for stromal VASH1 (red) and LVYE1 (green). Representative staining micrographs are shown (upper panel). T, tumor. Scale bar, 20 µm. Paired circulating exosomal miR-221-3p levels in CSCC patients were detected by qRT-PCR. Correlation analyses were calculated between two sets of quantified data as indicated (lower panel). **d** Illustrative model showing the mechanism whereby CSCC-secreted exosomal miR-221-3p promotes lymphangiogenesis by downregulating lymphatic VASH1 expression that then facilitates lymphatic metastasis. Error bars represent the mean ± SD of three independent experiments. * *P* < 0.05; ***, *P* < 0.001

To investigate whether cancer-secreted miR-221-3p can be detected in the circulation (peripheral blood) of CSCC patients, exosomes were isolated and characterized in the circulation of stage I–II CSCC patients with (*n* = 20) or without LN metastasis (*n* = 20). Typical morphology, size range, and associated proteins found in purified exosomes from the circulation were consistent with those from conditioned media (Fig. S8A–B). Moreover, circulating exosomal miR-221-3p expression was significantly higher in the circulation when isolated from LN-positive patients than from LN-negative patients (Fig. 6a). To further investigate whether circulating exosomal miR-221-3p in CSCC patients is functionally active in regulation of HLECs, we treated HLECs with circulating exosomes from LN-negative patients with low level miR-221-3p or LN-positive patients with high level miR-221-3p to perform a HLECs sprout assay. The results showed that circulating exosomes from LN-positive patients, but not LN-negative patients, increased sprouting lymphangiogenesis of HLECs, which could be abolished by yc=12?> miR-221-3p inhibitors (Fig. 6b).

In patients with paired circulation and tumor specimens, we found that circulating exosomal miR-221-3p level was positively associated with tumor miR-221-3p levels (*r* = 0.721, *P* < 0.001) and lymphangiogenesis (*r* = 0.512, *P* < 0.001) but was negatively correlated with VASH1 expression (*r* = − 0.588, *P* < 0.001) (Fig. 6c). Immunofluorescence and ISH in human CSCC tissues also demonstrated that high miR-221-3p levels in tumor were accompanied by reduced VASH1 expression in LYVE1-positive lymphatic vessels and enhanced PLVD. These observations further supported our conclusion that the CSCC-secreted exosomal miR-221-3p promoted lymphangiogenesis via downregulating VASH1 expression in HLECs and then facilitated lymphatic metastasis (Fig. 6d).

## Discussion

Tumor-induced peritumoral lymphangiogenesis has been reported to enhance the lymphatic dissemination of tumor cells to regional LNs [25]. The occurrence of regional LN metastasis at an early stage is considered to be a crucial step in CSCC progression [26]. Therefore, it is necessary to identify novel and effective biomarkers that discriminate between indolent and aggressive CSCC that can contribute to developing personalized diagnostic and therapeutic strategies for patients with different progression risks. Recently, circulating exosomal miRNAs have been recognized to be promising biomarkers for cancer patients owing to their profile of expression potentially reflecting dysregulated expression patterns in multiple human cancer types, as well as their non-invasive diagnostic approach and high stability in circulation [27, 28]. Here, our clinical evidence revealed that circulating exosomal miR-221-3p promoted lymphangiogenesis in vitro and was associated with PLVD and LN metastasis in patients with CSCC, suggesting that circulating exosomal miR-221-3p may serve as a diagnostic biomarker and therapeutic target for metastatic CSCC.

Many studies devoted to miRNAs expression profiling have shown significantly altered miRNAs signatures in various stages of cancer metastasis [29]. Our previous study also confirmed that higher miR-221-3p could be found in metastatic CSCC patients and it enhanced the malignancy of CSCC cells [14]. However, the metastasis cascade is not only dependent on miRNAs alternations within a subset of cancer cells but also involves interactions with surrounding cells that may significantly contribute to cancer progression [30]. Recently, cancer-secreted exosomal miRNAs have emerged as highly versatile regulators in this communication process [31]. Zhou et al.[32] reported that uptake of cancer-secreted miR-105 by vascular endothelial cells provoked vascular permeability and metastatic dissemination. In this study, we found that CSCC-secreted miR-221-3p could be horizontally transferred to LECs to promote lymphangiogenesis, thus facilitating lymphatic metastasis. Interestingly, cancer-secreted exosomes were able to directly transfer into LECs and other cells in LN (Fig. S9). The role of exosomal miRNAs in the tumor draining LNs is currently under investigation in our laboratory. Cancer-secreted miRNAs have also been reported to promote angiogenesis to accelerate tumor growth and metastasis [33]. Our study also found that miR-221-3p levels positively correlated with the MVD (Fig. S10). To exclude this confounding factor, we set up experiments to collect tumors at similar sizes after the indicated treatment to analyze the relationship among PLVD, microvascular density (MVD), and LN metastasis. Higher PLVD rather than MVD was positively correlated with LN metastasis (Fig. 4 and Fig. S5), implying that lymphangiogenesis may be more important than angiogenesis in CSCC LN metastasis. It is also intriguing why exosomal miR-221-3p does not affect LECs proliferation. A recent study by Kuehbacher et al. [34] found that non-specific silencing of miRNAs suppressed angiogenic activity, indicating the complexity of miRNAs-dependent regulation of angiogenesis. Similar to angiogenesis, lymphangiogenesis is also influenced by miRNAs [35]. We, therefore, concluded that CSCC-secreted miR-221-3p have an important role in a part of lymphangiogenesis as a result of increased migration and tube formation.

Various genes and pathways have been identified to be direct targets of miR-221-3p. For example, miR-221-3p targeted the JAK/STAT signaling pathway by directly inhibiting the expression of SOCS3 and IRF2 [36]. Here, we showed that VASH1, which was previously shown to negatively regulate lymphangiogenesis in our large predicted target cohorts, is of great interest as a downstream target of miR-221-3p, and our data confirmed that VASH1 was a novel direct target of miR-221-3p by luciferase reporter assay. Recognized as an endogenous angiogenesis inhibitor induced by vascular endothelial growth factor (VEGF) and fibroblast growth factor 2, VASH1 was proven to downregulate angiogenesis through a negative feedback mechanism under physiological conditions [37]. Nevertheless, this negative feedback system may be defective in tumor angiogenesis, as factors in the tumor microenvironment such as hypoxia and inflammation inhibit VASH1 expression in endothelial cells [38]. Considerable research suggested that knockdown of VASH1 might be a crucial driver of tumor lymphangiogenesis [22, 39]. Our results are consistent with these reports, namely, that re-expression and knockdown of VASH1 could, respectively, rescue and stimulate the effects of lymphangiogenesis induced by exosomal miR-221-3p in vitro. VASH1 was previously reported to be expressed in vascular endothelial cells but not in lymphatic endothelial cells in human lung cancer tissue [40], our data also revealed that reduced expression of lymphatic VASH1 accompanied by increased PLVD in CSCC specimens. There may be tissue-specific and cancer type-specific mechanisms for VASH1 expression in lymphatic cells. In this paper, we propose a new mechanism whereby CSCC-secreted miR-221-3p may promote lymphangiogenesis via downregulating VASH1.

It is noteworthy that exosomal miR-221-3p engage in transferring genetic information, whereas lymphangiogenic growth cytokines such as VEGF-C represents are small protein cytokines, and there are therefore two different interacting methods involved in manners for signal transmission between cancer cells and HLECs. Previous studies have demonstrated that VEGF-C signaling induced lymphangiogenesis via activation of the ERK and AKT pathways [24]. In our study, we found that HLECs treated with exosomes with high miR-221-3p led to an increase of phosphorylation of AKT and ERK1/2 proteins (Fig. S7A), which presents a potential mechanism for intercellular miR-221-3p-VASH1 axis induced lymphangiogenesis in CSCC. These results implied that the miR-221-3p-VASH1 axis and VEGF-C might share the same downstream pathways for lymphangiogenesis. Although miR-221-3p was reported to increase VEGF transcription in bladder cancer cells [41], our results found that miR-221-3p overexpression and knockdown did not induce VEGF-C mRNA or protein changes in CSCC cells (Fig. S7B). Considering the variations among different types of tumors, there may be tissue-specific mechanisms of VEGF-C regulation in response to miR-221-3p. Therefore, we considered that the miR-221-3p-VASH1 axis activated AKT and ERK signaling pathways and might work in a VEGF-C independent manner in HLECs. Developing personalized therapeutics, such as VEGF-C independent lymphangio-miR-221-3p that might serve as antilymphangiogenic targets in combination with current anti-VEGF-C therapies, seems a promising direction in the treatment of early-stage CSCC patients with LN metastasis.

In conclusion, our data provided evidence that high levels of circulating exosomal miR-221-3p are associated in CSCC with LN metastasis and are positively correlated with PLVD. Horizontal transfer of CSCC-secreted exosomal miR-221-3p into HLECs may promote lymphangiogenesis by regulating VASH1 signaling, which then promotes lymphatic metastasis. The newly identified intercellular miR-221-3p-VASH1 axis illustrated a critical molecular mechanism of CSCC progression and provided a novel diagnostic and therapeutic target for CSCC patients with LN metastasis.

## Materials and methods

### Cell lines

Human cervical squamous carcinoma cell lines Siha, Caski, C33a, MS751, ME180, and a non-carcinoma cervical epithelial HPV-16 E6/E7 transformed cell line, Ect1/E6E7 (Ect1), were all purchased from the ATCC and cultured according to their guidelines. HLECs and MLECs were respectively purchased from ScienCell and Cell Biologics and cultured in endothelial cell medium (ScienCell) with 5% fetal bovine serum (FBS; Gibco).

### Clinical specimens

CSCC specimens were obtained from voluntarily consenting patients without preoperative radiotherapy or chemotherapy at the Department of Gynecological Oncology of Nanfang Hospital (Guangzhou, PR China) between 2012 and 2014. The study was approved by the Institutional Research Ethics Committee. Detailed information on clinical specimens and research purposes are summarized in Supplementary Table S1–S2.

### Exosomes isolation and identification

A total of 10 ml cell conditioned medium or 250 µl serum was mixed with ExoQuick exosome precipitation solution and exosomes isolation was conducted according to the manufacturer’s protocol. After incubation overnight, the ExoQucik/biofluid mixture was centrifuged at 1500 × *g* for 30 min at 4 °C. The pelleted exosomes were subjected to electron microscope, protein assay, RNA extraction, in vitro treatment, or in vivo administration. For transmission electron microscopy (TEM), exosomes were fixed with 2% glutaraldehyde, loaded onto carbon-coated grids, and then negative-contrast stained with phosphotungstic acid. The grids were visualized by TEM (Hitachi). For protein assay, the exosome preparations used BCA Protein Assay Kit (Beyotime). For RNA extraction from exosomes, we used miRNeasy Mini Kit (Qiagen). For in vitro treatment, 10 µg of exosomes resuspended in 100 µl phosphate-buffered saline (PBS) were added to 1 × 10^5^ recipient cells for 48 h. For in vivo administration, 10 µg of exosomes resuspended in 20 µl PBS were injected into the center of the xenograft tumors twice a week. Purified exosomes were labeled with PKH67 (Sigma) as described previously [42].

### RNA extraction and qRT-PCR

RNA was extracted from cell lines, human CSCC tissues and tumor xenografts by TRIzol (Invitrogen). qRT-PCR was performed as previously described [43]. Specific primer sets for miR-221-3p and U6 were purchased from RiboBio Inc. The expression of miRNAs and mRNAs was normalized to U6 and GAPDH, respectively. The primer sequences are shown in supplementary Table S3.

### Immunohistochemistry

Tissue sections were subjected to IHC analysis as described previously [44]. The primary antibodies were as follows: anti-VASH1 (ab176114, Abcam), anti-CD31 (ab28364, Abcam), anti-LYVE1 (ab33682, Abcam), and anti-mCherry antibody (ab167453, Abcam). The secondary antibodies were horseradish peroxidase-conjugated anti-rabbit immunoglobulin-G antibody (ab6721, Abcam). Peritumoral lymphatic vessel density and micro-vessel density in tumor tissues were respectively determined by the number of LYVE1-positive vessels and CD31-positive vessels according to the methods described by Gombos [45].

### In situ hybridization

ISH was performed as described by Que [46].

### Staining assessment

The immunohistochemically and in situ hybridized stained tissue sections were reviewed and scored separately by two independent pathologists. For semi-quantitative evaluation of LYVE1 and miR-221-3p expression in tissue sections, a scoring system HSCORE was used as previously described [47]. HSCORE ≤ 2 was classified as low expression, and HSCORE > 2 was classified as high expression.

### Western blot

Western blot assay was performed as previously described [48]. The primary antibodies were as follows: anti-CD63 (ab68418, Abcam), anti-CD81(ab109201, Abcam), and anti-VASH1 (ab176114, Abcam); anti-phospho-AKT (9271, CST), anti-AKT (9272, CST), anti-phospho-ERK1/2 (4370, CST), anti-ERK1/2 (4695, CST), and anti-GAPDH antibody (2118, CST). The secondary antibodies were horseradish peroxidase-conjugated anti-rabbit immunoglobulin-G antibody (ab6721, Abcam).

### Immunofluorescence

Serial paraffin sections (4 µm) from human CSCC tissues were analyzed by immunofluorescence with the Opal 4-Color Kit (PerkinElmer) according to the manufacturer’s protocol. After deparaffinization, sections were microwaved in antigen retrieval buffer for 45 s at 100 °C, washed and blocked for 10 min at room temperature, followed by incubation with anti-VASH1 antibody (ab176114, Abcam). Horseradish peroxidase-conjugated secondary antibody was dropped onto slides for incubation for 10 min at room temperature. Subsequently, tyramide signal amplification (TSA) working buffer (Opal 570) was used to amplify the signal on slides. After eliminating anti-VASH1 and secondary antibodies by microwaving, the above procedures were repeated with anti-LYVE1 antibody (ab33682, Abcam) and TSA working buffer (Opal 520). Sections were mounted in neutral gum and visualized by a fluorescence microscope (Olympus). The fluorescence intensity of VASH1 expression was analyzed by ImageJ software.

### Stable transfection with lentiviral vector

Lenti-mecherry containing an miR-221-3p overexpression sequence and its negative control RNA (miR-NC), or containing an miR-221-3p knockdown segment and its negative control vector (anti-NC) were all purchased from GeneChem Inc. Ect1 and Siha cells were transfected with lenti-mCherry/miR-221-3p and stably expressing mCherry fluorescent protein signals were selected for further experiments by flow cytometer.

### Transient transfection with oligonucleotides and plasmids

The miR-221-3p mimic and its NC were designed and cloned by RiboBio Inc. The VASH1-coding sequence (without 3′-UTR) was cloned into pCDNA3.1( + )-Vector (Invitrogen). The empty vector was used as a blank control. siVASH1 and its NC siRNA were designed and synthesized by GenePharma Inc. Lipofectamine 2000 Reagent (Invitrogen) was then used to transfect miR-221-3p mimic, siVASH1, and pCDNA3.1( + )-VASH1 according to the manufacturer’s protocol. For RNA extraction, western blot and in vitro functional assays, cells were used 48 h after transfection. The sequence of siVASH1 and siRNA are shown in supplementary Table S3.

### Luciferase reporter assay

The expression of miR-221-3p targeted gene was measured by using a dual-luciferase reporter assay in 293 T cells. The putative miR-221-3p complementary site in the 3′-UTR of VASH1 or its mutant sequence was cloned into the pmiR-RB-REPORT vector (RiboBio Inc.). Then, pmiR-RB-REPORT-VASH1-3′-UTR-WT or pmiR-RB-REPORT-VASH1-3′-UTR-MT were co-transfected into 293 T cells with miR-221-3p mimic or its NC in 48-well plates, collected 48 h after transfection and analyzed by using a Dual-Luciferase Reporter Assay System (Promega). Firefly luciferase signal was used for normalization.

### Bioinformatic miRNAs target prediction

Three online programs TargetScan, miRWalk, and PicTar were used to predict potential target genes for miR-221-3p. The Gene Ontology Consortium was used to identify negative regulators of lymphangiogenesis.

### HLECs tube formation assay

HLECs tube formation assay was performed as described in our previous study [44].

### HLECs sprouting assay

HLECs were coated on microcarrier-beads (Sigma) in a ratio of 10^6^ cells per 2000 beads and incubated for 4 h at 37 °C. After culture in a six-well dish for 24 h, coated beads were embedded in a solution of fibrinogen in EGM2 medium, and then overlaid with normal human lung fibroblasts in a 24-well dish. Images were captured under a phase contrast microscope (Olympus).

### Transwell migration assay

In total, 1 × 10^5^ cells in 200 µl 1640 medium without FBS were seeded on a fibronectin-coated polycarbonate membrane insert in a Transwell apparatus (Corning). In the lower chamber, 600 µl 1640 with 10% FBS was added as a chemoattractant. After the cells that invaded to the bottom of the insert membrane were fixed with methanol, the insert was stained with Giemsa (Sigma) and we counted the cell numbers under a microscope in five random fields ( × 200).

### Cell counting kit-8 (CCK-8) assay

CCK-8 assay was performed as described previously [49].

### Popliteal LN metastasis model

Female nude mice (4 weeks old) were purchased from the Experimental Animal Center, Southern Medical University (Guangzhou, PR China). The studies were approved by the Institutional Animal Research Ethics Committee of Southern Medical University. Siha/anti-221-3p cells (5 × 10^6^) stably expressing mCherry were injected into the footpads of the mice. Tumor size (mm^3^) was measured every 4 days and calculated by the formula: volume = (width)^2^ × length/2. The mice were killed when the primary tumors reached a comparable size of ~ 150 mm^3^, then the popliteal LNs were paraffin embedded and analyzed for mCherry expression by IHC with anti-mCherry antibody (Abcam). Positive LNs were identified by detecting mCherry staining under a Nikon upright microscope. The ratio of metastasis-positive to total dissected popliteal LN was calculated.

### ELISA

VEGF-C in the culture supernatants of tumor cells was quantified using a human VEGF-C ELISA Kit (eBioscience) according to the manufacturer’s protocol.

### Statistical analysis

SPSS V.13.0 software was used for statistical analysis. Data are expressed as the mean ± standard deviation. One-way analysis of variance was used for comparisons among groups. The *χ*^2^-test was applied for categorical variables. Correlation analysis was performed using the Spearman rank test. Differences were considered to be statistically significant when *P* < 0.05.

## Electronic supplementary material

Supplementary Information related to manuscript
