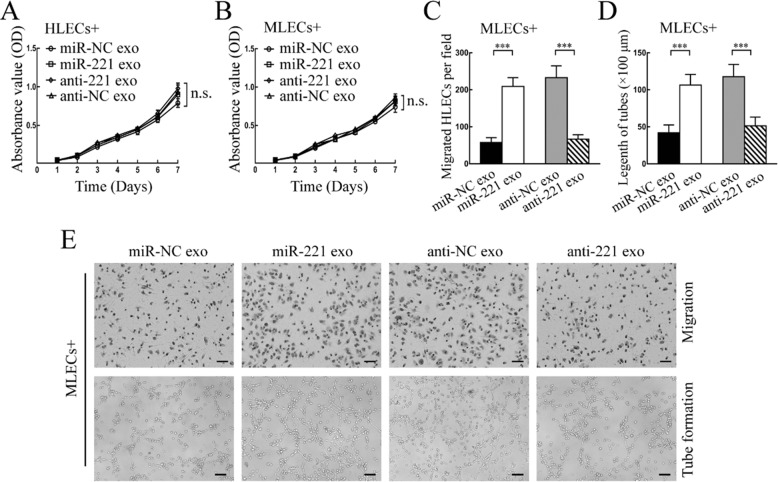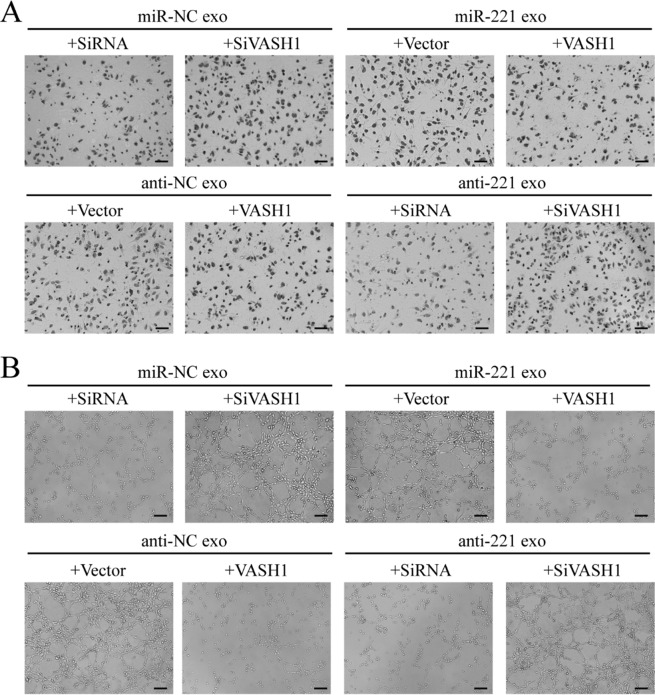# Correction to: Cervical squamous cell carcinoma-secreted exosomal miR-221-3p promotes lymphangiogenesis and lymphatic metastasis by targeting VASH1

**DOI:** 10.1038/s41388-021-02165-x

**Published:** 2022-01-19

**Authors:** Chen-Fei Zhou, Jing Ma, Lei Huang, Hong-Yan Yi, Yan-Mei Zhang, Xiang-Guang Wu, Rui-Ming Yan, Li Liang, Mei Zhong, Yan-Hong Yu, Sha Wu, Wei Wang

**Affiliations:** 1 Department of Obstetrics and Gynecology, The First Affiliated Hospital of Guangzhou Medical University, Guangzhou, 510120 China; 2 Department of Obstetrics and Gynecology, Nanfang Hospital/The First School of Clinical Medicine, Southern Medical University, Guangzhou, 510515 China; 3 Institute of Cellular Medicine, Faculty of Medical Sciences, Newcastle University, Framlington Place, Newcastle-Upon-Tyne, NE2 4HH UK; 4 Department of Immunology, School of Basic Medical Sciences, Southern Medical University, Guangdong Provincial Key Laboratory of Proteomic, Guangzhou, 510515 China; 5 Department of Pathology, School of Basic Medical Sciences, Southern Medical University, Guangzhou, 510515 China

**Keywords:** Cervical cancer, Metastasis

Correction to: *Oncogene* (2019) 38:1256–1268 10.1038/s41388-018-0511-x, published online 25 September 2018

After the publication of this article, the authors noted errors in Supplementary Figs. 2 and 6.

In Supplementary Fig. 2, panel E, the same image was mistakenly presented for both miR-221 exo and anti-NC exo.

In Supplementary Fig. 6, panel A, the incorrect image was presented to show anti-221 exo plus SiRNA.

The authors confirm that these errors do not alter the conclusions of the paper.

The corrected figures are given below: