# Supplementary material for: Cervical squamous cell carcinoma-secreted exosomal miR-221-3p promotes lymphangiogenesis and lymphatic metastasis by targeting VASH1
Source: Oncogene. 2018 Sep 25;38(8):1256–68. doi: 10.1038/s41388-018-0511-x (PMC6363643; doi:10.1038/s41388-018-0511-x)
Supplement: Supplementary file 1 — Supplementary Information related to manuscript [file 41388_2018_511_MOESM1_ESM.docx]

**Supplementary Information**

**Supplementary Figures**


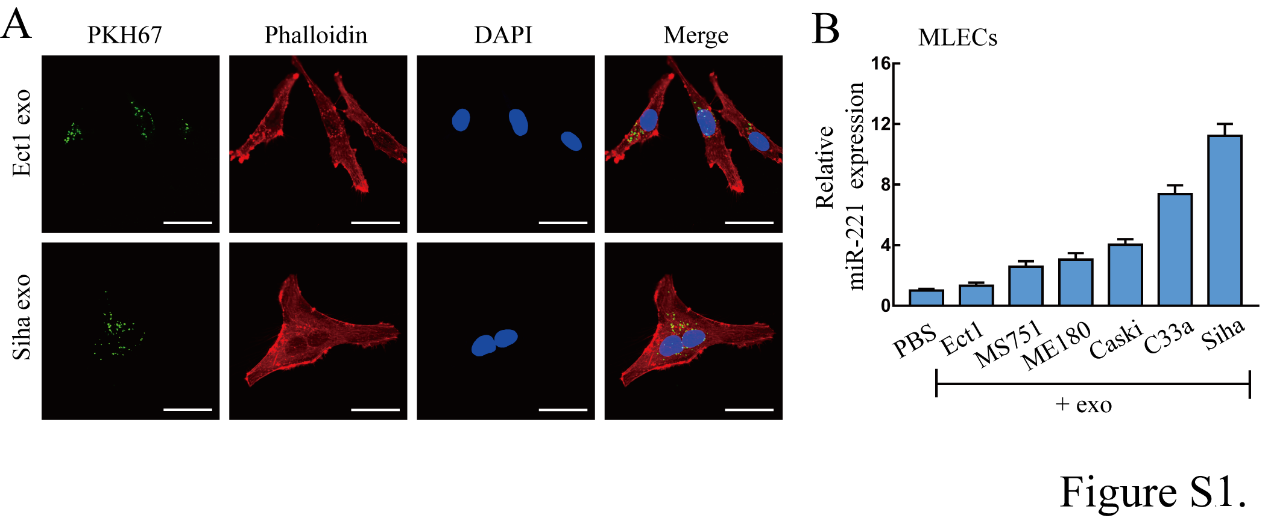


**Figure S1. CSCC-secreted miR-221-3p can be transferred to MLECs via exosomes, related to Fig. 2.** A. Mouse lymphatic endothelial cells (MLECs) pre-treated with PKH67-labeled exosomes secreted by Ect1 and Siha for 24h were stained by phalloidin (red) and DAPI (blue) for confocal microscopy analysis. Scale bar, 20 µm. B. miR-221-3p levels in MLECs pre-treated with PBS or the indicated exosomes for 24h were detected by qRT-PCR. Exo, exosomes. miR-221, hsa-miR-221-3p. Error bars represent the mean ± SD of three independent experiments.





**Figure S2. Effect of CSCC-secreted exosomal miR-221-3p on MLECs is the same as that of HLECs, related to Fig. 3.** A. CCK-8 proliferation assay in HLECs pre-treated with the indicated exosomes. B. CCK-8 proliferation assay in MLECs pre-treated with the indicated exosomes. C. Transwell migration assay in MLECs pre-treated with the indicated exosomes. Average migrated cells per field were calculated. D. Tube formation assay in MLECs pre-treated with the indicated exosomes. Average length of tubes per field were calculated. E. Representative micrographs of migration (upper panel) and tube formation assays (lower panel) in MLECs pre-treated with indicated exosomes are shown. Scale bar, 50 µm. Error bars represent the mean ± SD of three independent experiments. n.s., not significant; ***, P<0.001.


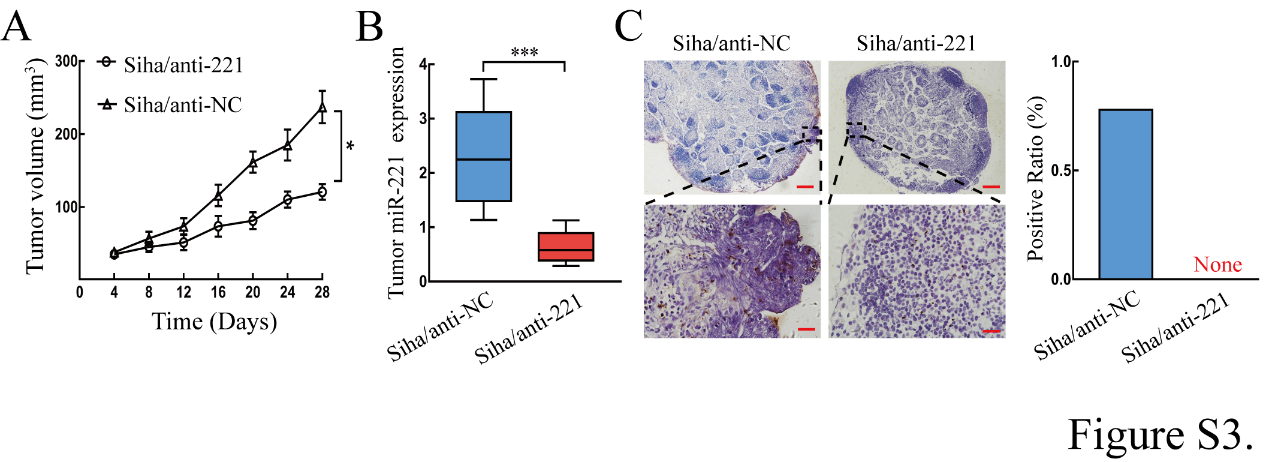


**Figure S3. Siha/anti-221-3p exhibits poor metastatic capacity in LN, related to Fig. 4.** A popliteal lymph node metastasis model was established in nude mice by inoculating the footpad with Siha/anti-NC or Siha/anti-221-3p (n=3/group, repeated twice) stably expressing mCherry. A. Growth curve of footpad tumors performed by Siha/anti-NC and Siha/anti-221-3p. B. miR-221-3p levels in footpad tumors were detected by qRT-PCR. C. Staining of mCherry in popliteal LNs from mice treated with Siha/anti-NC or Siha/anti-221-3p. Representative micrographs are shown (left). Metastasis-positive LNs were identified by staining for cancer cell-expressed mCherry. The ratio of metastasis-positive to total dissected popliteal LN was calculated (right). Scale bar, upper panel, 200 µm; lower panel, 20 µm. Error bars represent the mean ± SD of three independent experiments. *, P<0.05. ***, P<0.001.


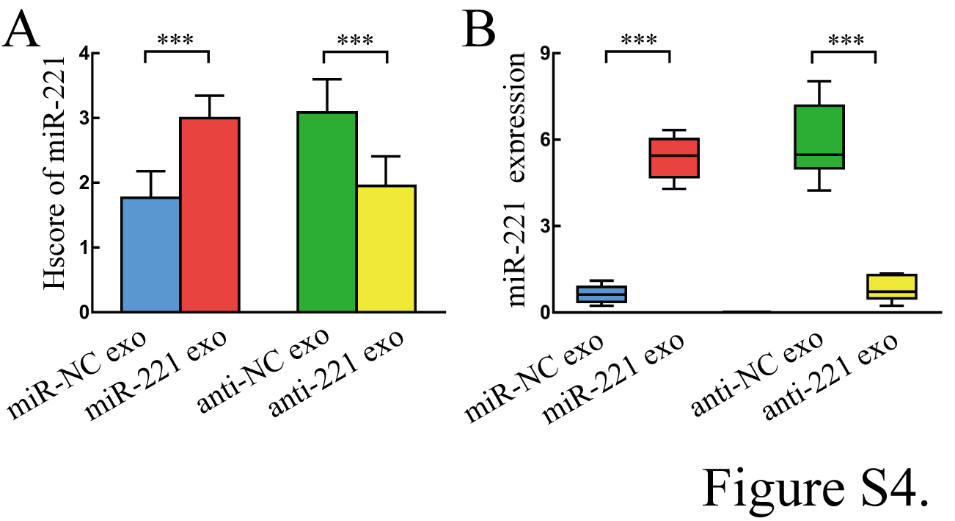


**Figure S4. miR-221-3p expression in primary footpad tumors treated with the indicated exosomes, related to Fig. 4.** A. miR-221-3p levels were detected by ISH. B. miR-221-3p levels were detected by qRT-PCR. Error bars represent the mean ± SD of three independent experiments. ***, P<0.001.


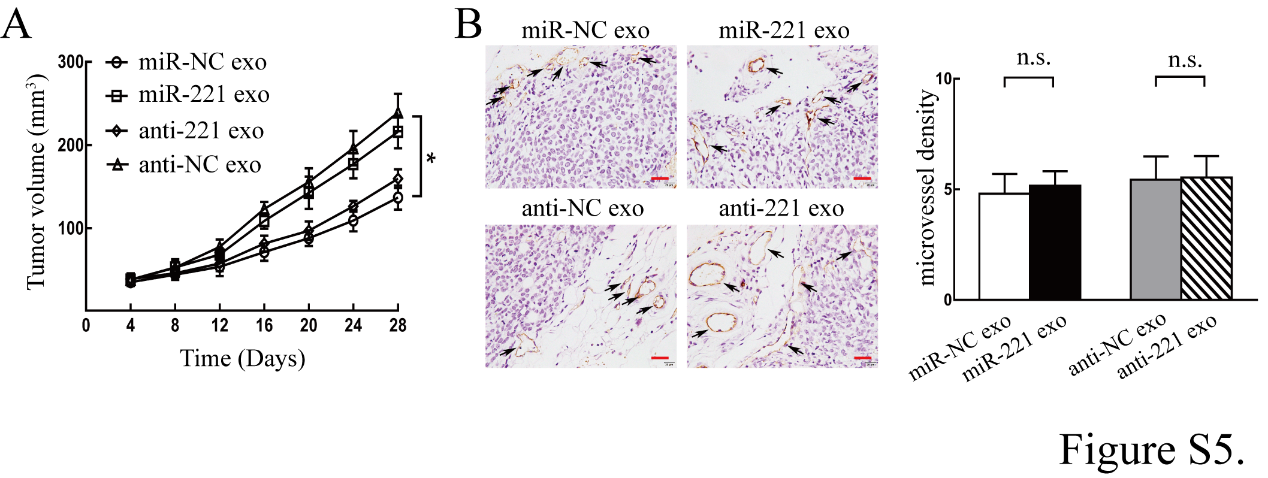


**Figure S5. There was no significant difference in** **angiogenesis at a comparable tumor size (150 mm^3^) when treated with the indicated exosomes, related to Fig. 4.** A. Growth curve of footpad tumors treated with the indicated exosomes (n=3/group, repeated twice). B. Micro-vessels were visualized by staining for CD31 in footpad tumors treated with the indicated exosomes. Representative micrographs of positive staining are shown (left). Micro-vessel density was quantified (right). Scale bar, 50 µm. Error bars represent the mean ± SD of three independent experiments. n.s., not significant; *, P<0.05.





**Figure S6. CSCC-secreted exosomal miR-221-3p directly targets VASH1 to induce lymphangiogenesis in HLECs, related to Fig. 5.** A. Representative images of transwell migration assays in HLECs treated as indicated are shown. Scale bar, 50 µm. B. Representative images of Transwell migration assays in HLECs treated as indicated are shown. Scale bar, 100 µm.


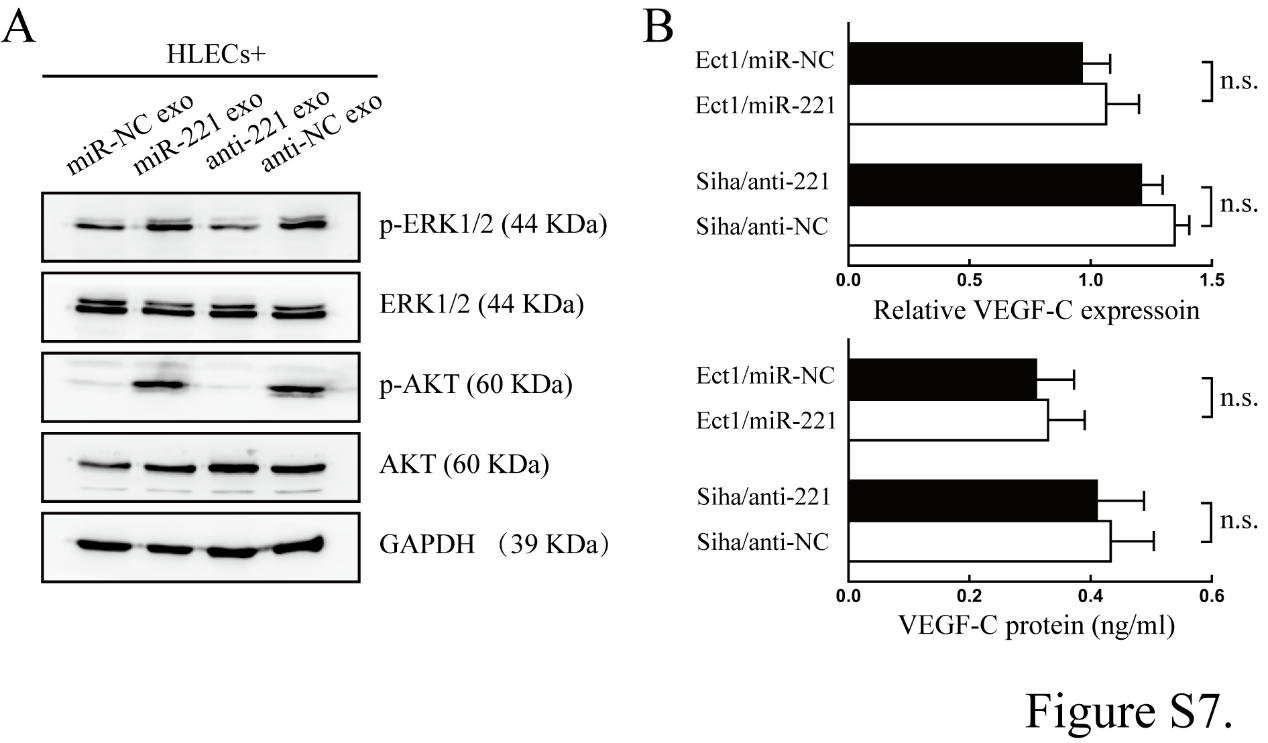


**Figure S7. CSCC-secreted exosomal miR-221-3p activated ERK and AKT pathways in HLECs independent of VEGF-C**. A. ERK and AKT signaling pathways in HLECs pre-treated indicated exosomes were detected by western blot. B. mRNA and protein levels of VEGF-C were detected in miR-221-3p-overexpressing Ect1 and miR-221-3p-knockdown Siha compared with the negative control (NC) by qRT-PCR (upper panel) and ELISA (lower panel). Error bars represent the mean ± SD of three independent experiments. n.s., not significant.


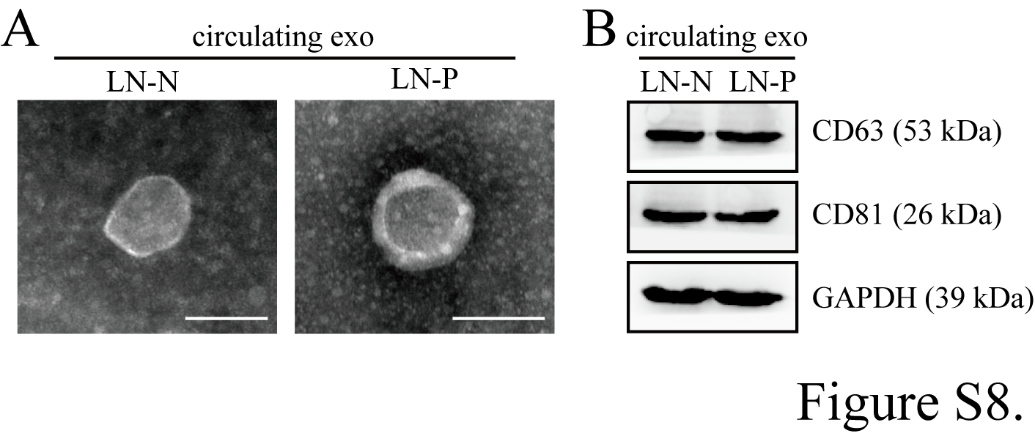


**Figure S8. Identification of circulating exosomes from CSCC patients with negative LNs (LN-N; n=20) and positive LNs (LN-P; n=20), related to Fig. 6.** A. TEM images of circulating exosomes are shown. Scale bar, 50 nm. B. Western blot analysis of exosomal markers CD63 and CD81.

**
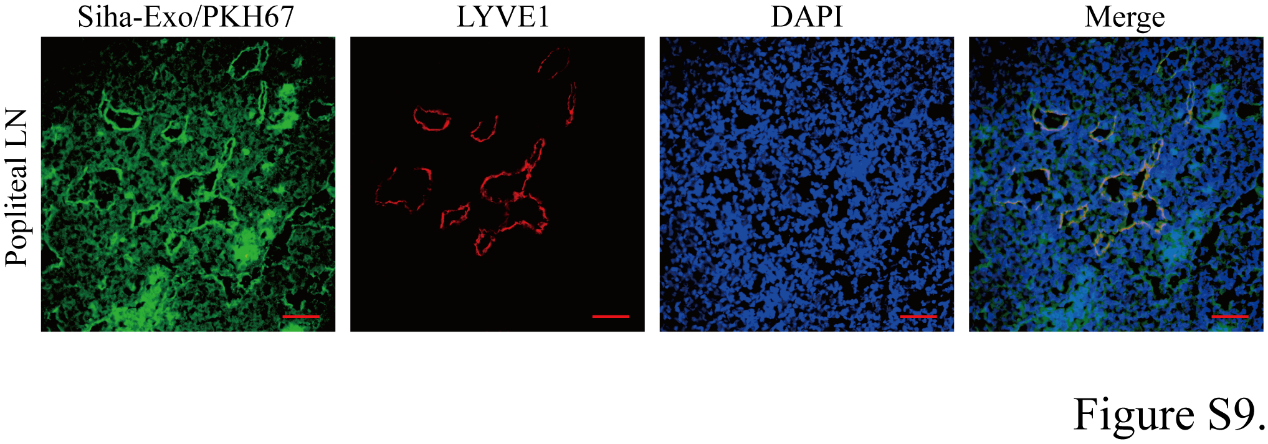
**

**Figure S9. Representative micrographs of Siha-Exo transferred into LECs from popliteal LN are shown.** Siha-Exo was labeled with PKH67 (green) and injected into footpad. Popliteal LNs were collected after 48 hours and LEC were immune-fluorescence stained on frozen section for with LYVE1 (red). Scale bar, 20 µm.

**
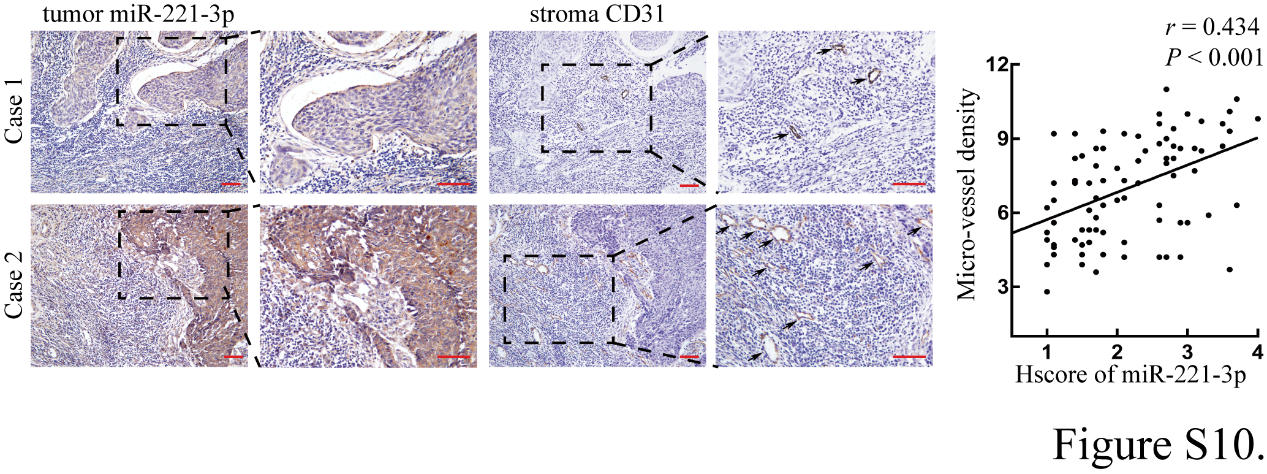
**

**Fig S10. Staining of tumor miR-221-3p and stromal CD31 (vascular marker) in serial sections of CSCC specimens**. Representative micrographs are shown (left). The CD31 positive micro-vessels are indicated by black arrows. Correlations between miR-221-3p staining and micro-vessel density were analyzed (right). Scale bar, 50 µm.

**Supplementary Tables**

**Table S1. Clinical characteristics of patients in the tumor study, related to Fig 1.**

| Patient | Age | HC-II | Differentiation | Histology | Stage | Progression |
| --- | --- | --- | --- | --- | --- | --- |
| 1 | 41 | + | Moderately | Squamous | IB1 | N |
| 2 | 42 | + | Moderately | Squamous | IB1 | N |
| 3 | 49 | + | Moderately | Squamous | IIB | N |
| 4 | 70 | + | Moderately | Squamous | IIB | N |
| 5 | 54 | + | Well | Squamous | IB2 | N |
| 6 | 52 | + | Moderately | Squamous | IB1 | N |
| 7 | 52 | + | Moderately | Squamous | IB2 | N |
| 8 | 47 | + | Poorly | Squamous | IB1 | N |
| 9 | 43 | + | Moderately | Squamous | IB1 | N |
| 10 | 39 | + | Poorly | Squamous | IB1 | N |
| 11 | 46 | + | Moderately | Squamous | IB1 | N |
| 12 | 40 | + | Well | Squamous | IIA1 | N |
| 13 | 51 | + | Moderately | Squamous | IB1 | N |
| 14 | 55 | + | Moderately | Squamous | IB1 | N |
| 15 | 59 | + | Moderately | Squamous | IB2 | N |
| 16 | 44 | + | Moderately | Squamous | IB1 | N |
| 17 | 35 | + | Poorly | Squamous | IIB | N |
| 18 | 42 | + | Moderately | Squamous | IIB | N |
| 19 | 57 | + | Moderately | Squamous | IB1 | N |
| 20 | 58 | + | Moderately | Squamous | IIB | N |
| 21 | 51 | + | Moderately | Squamous | IIB | N |
| 22 | 50 | + | Poorly | Squamous | IB1 | N |
| 23 | 60 | + | Moderately | Squamous | IB1 | N |
| 24 | 52 | + | Moderately | Squamous | IIA1 | N |
| 25 | 56 | + | Moderately | Squamous | IIA1 | N |
| 26 | 42 | + | Poorly | Squamous | IB1 | N |
| 27 | 36 | + | Moderately | Squamous | IB2 | N |
| 28 | 33 | + | Moderately | Squamous | IB1 | N |
| 29 | 38 | + | Poorly | Squamous | IB1 | N |
| 30 | 43 | + | Poorly | Squamous | IB1 | N |
| 31 | 37 | + | Poorly | Squamous | IB1 | N |
| 32 | 48 | + | Moderately | Squamous | IIA1 | N |
| 33 | 45 | + | Moderately | Squamous | IB1 | N |
| 34 | 47 | + | Moderately | Squamous | IB1 | N |
| 35 | 45 | + | Moderately | Squamous | IIA1 | N |
| 36 | 48 | + | Moderately | Squamous | IIB | N |
| 37 | 36 | + | Moderately | Squamous | IIB | N |
| 38 | 42 | + | Moderately | Squamous | IB1 | N |
| 39 | 50 | + | Moderately | Squamous | IIA2 | N |
| 40 | 62 | + | Moderately | Squamous | IIB | N |
| 41 | 29 | + | Moderately | Squamous | IB1 | N |
| 42 | 64 | + | Poorly | Squamous | IIA1 | N |
| 43 | 48 | + | Moderately | Squamous | IIA1 | N |
| 44 | 57 | + | Moderately | Squamous | IIA2 | N |
| 45 | 60 | + | Well | Squamous | IIA1 | N |
| 46 | 34 | + | Moderately | Squamous | IIB | N |
| 47 | 51 | + | Moderately | Squamous | IB1 | N |
| 48 | 42 | + | Moderately | Squamous | IB1 | N |
| 49 | 62 | + | Poorly | Squamous | IB1 | N |
| 50 | 67 | + | Well | Squamous | IB1 | N |
| 51 | 51 | + | Poorly | Squamous | IIA1 | N |
| 52 | 61 | + | Moderately | Squamous | IIB | N |
| 53 | 62 | + | Moderately | Squamous | IB1 | N |
| 54 | 49 | + | Moderately | Squamous | IIA1 | N |
| 55 | 49 | + | Moderately | Squamous | IIA2 | N |
| 56 | 37 | + | Moderately | Squamous | IB1 | N |
| 57 | 49 | + | Well | Squamous | IA2 | N |
| 58 | 51 | + | Moderately | Squamous | IB1 | N |
| 59 | 44 | + | Moderately | Squamous | IB1 | N |
| 60 | 42 | + | Moderately | Squamous | IB2 | N |
| 61 | 57 | + | Moderately | Squamous | IIA1 | N |
| 62 | 45 | + | Well | Squamous | IB2 | N |
| 63 | 51 | + | Poorly | Squamous | IB1 | N |
| 64 | 61 | + | Moderately | Squamous | IIA1 | N |
| 65 | 44 | + | Poorly | Squamous | IIA1 | Y |
| 66 | 58 | + | Moderately | Squamous | IB1 | Y |
| 67 | 29 | + | Poorly | Squamous | IIB | Y |
| 68 | 41 | + | Moderately | Squamous | IIB | Y |
| 69 | 49 | + | Poorly | Squamous | IB1 | Y |
| 70 | 39 | + | Moderately | Squamous | IB1 | Y |
| 71 | 72 | + | Moderately | Squamous | IIB | Y |
| 72 | 55 | + | Poorly | Squamous | IIA1 | Y |
| 73 | 53 | + | Moderately | Squamous | IB1 | Y |
| 74 | 48 | + | Moderately | Squamous | IB2 | Y |
| 75 | 45 | + | Moderately | Squamous | IIA2 | Y |
| 76 | 51 | + | Moderately | Squamous | IIA2 | Y |
| 77 | 46 | + | Poorly | Squamous | IIB | Y |
| 78 | 59 | + | Moderately | Squamous | IIB | Y |
| 79 | 42 | + | Moderately | Squamous | IB2 | Y |
| 80 | 56 | + | Moderately | Squamous | IIA2 | Y |
| 81 | 62 | + | Moderately | Squamous | IIA2 | Y |
| 82 | 43 | + | Moderately | Squamous | IB1 | Y |
| 83 | 61 | + | Moderately | Squamous | IIA2 | Y |
| 84 | 58 | + | Poorly | Squamous | IB1 | Y |
| 85 | 33 | + | Moderately | Squamous | IB1 | Y |
| 86 | 66 | + | Moderately | Squamous | IIA1 | Y |
| 87 | 44 | + | Moderately | Squamous | IIB | Y |
| 88 | 39 | + | Moderately | Squamous | IB2 | Y |
| 89 | 38 | + | Moderately | Squamous | IB1 | Y |
| 90 | 34 | + | Moderately | Squamous | IIB | Y |
| 91 | 50 | + | Poorly | Squamous | IIA2 | Y |
| 92 | 55 | + | Moderately | Squamous | IIB | Y |
| 93 | 48 | + | Moderately | Squamous | IB2 | Y |
| 94 | 42 | + | Moderately | Squamous | IB1 | Y |
| 95 | 34 | + | Moderately | Squamous | IIA2 | Y |
| 96 | 39 | + | Moderately | Squamous | IIB | Y |
| 97 | 47 | + | Moderately | Squamous | IIB | Y |
| 98 | 48 | + | Moderately | Squamous | IIA2 | Y |
| 99 | 46 | + | Poorly | Squamous | IIB | Y |
| 100 | 49 | + | Moderately | Squamous | IB1 | Y |
| 101 | 34 | + | Poorly | Squamous | IIA1 | Y |
| 102 | 62 | + | Poorly | Squamous | IB1 | Y |
| 103 | 46 | + | Moderately | Squamous | IB1 | Y |
| 104 | 57 | + | Moderately | Squamous | IIA1 | Y |
| 105 | 46 | + | Moderately | Squamous | IIB | Y |
| 106 | 43 | + | Poorly | Squamous | IIA1 | Y |
| 107 | 40 | + | Moderately | Squamous | IIB | Y |

**Table S2. Clinical characteristics of patients in the circulation study, related to Fig 7.**

| Patient | Age | HC-II | Differentiation | Histology | Stage | Progression |
| --- | --- | --- | --- | --- | --- | --- |
| 1 | 66 | + | Moderately | Squamous | IB1 | N |
| 2 | 53 | + | Well | Squamous | IA1 | N |
| 3 | 41 | + | Moderately | Squamous | IB1 | N |
| 4 | 45 | + | Poorly | Squamous | IB2 | N |
| 5 | 60 | + | Moderately | Squamous | IA1 | N |
| 6 | 44 | + | Poorly | Squamous | IB2 | N |
| 7 | 39 | + | Moderately | Squamous | IB1 | N |
| 8 | 43 | + | Moderately | Squamous | IIB | N |
| 9 | 38 | + | Moderately | Squamous | IB1 | N |
| 10 | 52 | + | Poorly | Squamous | IIB | N |
| 11 | 42 | + | Moderately | Squamous | IIB | N |
| 12 | 64 | + | Moderately | Squamous | IB1 | N |
| 13 | 49 | + | Moderately | Squamous | IIA1 | N |
| 14 | 42 | + | Well | Squamous | IIA2 | N |
| 15 | 61 | + | Poorly | Squamous | IB1 | N |
| 16 | 57 | + | Moderately | Squamous | IB2 | N |
| 17 | 44 | + | Moderately | Squamous | IA2 | N |
| 18 | 37 | + | Moderately | Squamous | IIB | N |
| 19 | 45 | + | Poorly | Squamous | IIB | N |
| 20 | 39 | + | Moderately | Squamous | IA2 | N |
| 21 | 57 | + | Poorly | Squamous | IIB | Y |
| 22 | 44 | + | Moderately | Squamous | IB2 | Y |
| 23 | 39 | + | Poorly | Squamous | IIB | Y |
| 24 | 36 | + | Moderately | Squamous | IIB | Y |
| 25 | 47 | + | Poorly | Squamous | IB2 | Y |
| 26 | 59 | + | Moderately | Squamous | IIB | Y |
| 27 | 61 | + | Poorly | Squamous | IB1 | Y |
| 28 | 46 | + | Moderately | Squamous | IIB | Y |
| 29 | 60 | + | Moderately | Squamous | IB2 | Y |
| 30 | 41 | + | Poorly | Squamous | IIA1 | Y |
| 31 | 47 | + | Moderately | Squamous | IIA2 | Y |
| 32 | 63 | + | Moderately | Squamous | IIB | Y |
| 33 | 52 | + | Moderately | Squamous | IIA2 | Y |
| 34 | 53 | + | Moderately | Squamous | IIB | Y |
| 35 | 57 | + | Moderately | Squamous | IB2 | Y |
| 36 | 53 | + | Poorly | Squamous | IB1 | Y |
| 37 | 67 | + | Moderately | Squamous | IIB | Y |
| 38 | 56 | + | Poorly | Squamous | IIA2 | Y |
| 39 | 42 | + | Moderately | Squamous | IIB | Y |
| 40 | 49 | + | Moderately | Squamous | IB1 | Y |

**Table S3. Detailed primer sequences in the study.**

|  | Forward | Reverse |
| --- | --- | --- |
| VASH1 | CATGGGAGGGCTTGATGAAGG | CAAGGTCAGCATGGACTAGGC |
| VEGF-C | ATGTTTTCCTCGGATGCTGGA | CATTGGCTGGGGAAGAGTTT |
| GAPDH | CCATCAATGACCCCTTCATTGACC | GAAGGCCATGCCAGTGAGCTTCC |
| SiVASH1 | GCCGUGAUCCUGGGAAUUUTT | AAAUUCCCAGGAUCACGGCTT |
| SiRNA | UGACCUCAACUACAUGGUUTT | AACCAUGUAGUUGAGGUCATT |

**Table S4. Target genes list of miR-221-3p predicted by multiple algorithms (miRWalk, PicTar and TargetScan). Provided as an excel spreadsheet file.**

**Table S5. Negative regulators of lymphangiogenesis confirmed by GOC.**

| No. | Symbol | Object Name |
| --- | --- | --- |
| 1 | Epha2 | Eph receptor A2 |
| 2 | FOXC1 | Forkhead Box C1 |
| 3 | VASH1 | Vasohibin 1 |
